# Autolysosomal Dysfunction in Obesity-induced Metabolic Inflammation and Related Disorders

**DOI:** 10.1007/s13679-025-00638-8

**Published:** 2025-05-14

**Authors:** Lenny Yi Tong Cheong, Eka Norfaishanty Saipuljumri, Gavin Wen Zhao Loi, Jialiu Zeng, Chih Hung Lo

**Affiliations:** 1 Lee Kong Chian School of Medicine, Nanyang Technological University, Singapore, 308232 Singapore; 2 Program in Neuroscience & Cognitive Science, University of Arizona, Tucson, AZ 85721 USA; 3 School of Biomedical Sciences, The University of Queensland, St Lucia, QLD 4072 Australia; 4 Department of Biomedical and Chemical Engineering, Syracuse University, Syracuse, NY 13244 USA; 5 Interdisciplinary Neuroscience Program, Syracuse University, Syracuse, NY 13244 USA; 6 Department of Biology, Syracuse University, Syracuse, NY 13244 USA

**Keywords:** Metabolic inflammation, Autophagy function, Lysosomal acidification, Neuroinflammation, Neurodegeneration, Therapeutic strategies

## Abstract

**Purpose of Review:**

Obesity is a global health crisis affecting individuals across all age groups, significantly increasing the risk of metabolic disorders such as type 2 diabetes (T2D), metabolic dysfunction-associated fatty liver disease (MAFLD), and cardiovascular diseases. The World Health Organization reported in 2022 that 2.5 billion adults were overweight, with 890 million classified as obese, emphasizing the urgent need for effective interventions. A critical aspect of obesity’s pathophysiology is meta-inflammation—a chronic, systemic low-grade inflammatory state driven by excess adipose tissue, which disrupts metabolic homeostasis. This review examines the role of autolysosomal dysfunction in obesity-related metabolic disorders, exploring its impact across multiple metabolic organs and evaluating potential therapeutic strategies that target autophagy and lysosomal function.

**Recent Findings:**

Emerging research highlights the importance of autophagy in maintaining cellular homeostasis and metabolic balance. Obesity-induced lysosomal dysfunction impairs the autophagic degradation process, contributing to the accumulation of damaged organelles and toxic aggregates, exacerbating insulin resistance, lipotoxicity, and chronic inflammation. Studies have identified autophagic defects in key metabolic tissues, including adipose tissue, skeletal muscle, liver, pancreas, kidney, heart, and brain, linking autophagy dysregulation to the progression of metabolic diseases. Preclinical investigations suggest that pharmacological and nutritional interventions—such as AMPK activation, caloric restriction mimetics, and lysosomal-targeting compounds—can restore autophagic function and improve metabolic outcomes in obesity models.

**Summary:**

Autolysosomal dysfunction is a pivotal contributor to obesity-associated metabolic disorders , influencing systemic inflammation and metabolic dysfunction. Restoring autophagy and lysosomal function holds promise as a therapeutic strategy to mitigate obesity-driven pathologies. Future research should focus on translating these findings into clinical applications, optimizing targeted interventions to improve metabolic health and reduce obesity-associated complications.

## Introduction

Obesity is an increasing global health concern in children, adolescence and adults, and can lead to the development of metabolic disorders including type 2 diabetes (T2D), metabolic dysfunction-associated steatotic liver disease (MASLD), and cardiovascular diseases [1, 2]. As stated by the World Health Organization (WHO), in 2022, 2.5 billion adults (18 years and older) were overweight; of these, 890 million were living with obesity and it is a challenge to public health. Excess dietary intake and reduced activity levels contribute to the growing epidemic of obesity. Current treatments primarily aim to reduce caloric intake, but their effectiveness is limited [3]. To improve obesity management and its associated complications, a deeper understanding of obesity's pathophysiology is crucial [3]. Over the past 2 decades, it has been recognized that obesity is associated with chronic low-grade inflammation or metabolic inflammation in a variety of tissues, including adipose tissue (AT), skeletal muscle, liver, pancreas islet, heart, kidney and the brain [4, 5, 6].

Autophagy plays a key role in maintaining cellular homeostasis and organ function by removing accumulated proteins, lipids, and organelles, which may induce toxicity to the cells [7]. There are mainly three forms of autophagy: macroautophagy, chaperone-mediated autophagy, and microautophagy. The most well-studied form of autophagy is macroautophagy, herein referred to as autophagy, which begins where bulk cytoplasmic material is sequestered by a double-membrane structure termed as autophagosomes [7], which can be determined by visualization of the LC3-II protein because of its localization on the autophagosome membrane [8]. Subsequently, the autophagosome fuses with lysosomes leading to the degradation and recycling of the encapsulated cargo [7].The encapsulated cargo can vary from protein aggregates (e.g., amyloid beta, tau, alpha synuclein) [9], lipid metabolites [10] or damaged organelles (e.g., mitochondria, endoplasmic reticulum) [11, 12]. A crucial part to controlling autophagic degradative process lies in the lysosomes, which is the last step of the autophagic process where the fusion of acidic lysosomes with autophagosomes allows the degradation of the encapsulated waste cellular contents [7]. Maintenance of the acidic environment is mainly through the lysosomal V-ATPase, which actively transports H^+^ ions from the cytosol into the lysosomal lumen [13]. This acidic environment is essential for the optimal activity of hydrolytic enzymes—such as proteases, lipases, and nucleases—that degrade complex macromolecules into their fundamental components [13]. Defective autolysosomal function has been shown to result in accumulation of lipid droplets and impaired mitochondrial function [14]. Furthermore, lysosomal and autophagy dysfunction has been shown to be a trigger for inflammation in obesity [15, 16], which can further propagate systemic inflammation and exacerbate disease progression. Consequently, dysregulated autolysosomal function play important roles in mediating obesity-induced metabolic disorders [3, 17], including T2D [18], MASLD [14], chronic kidney diseases (CKD) [19], atherosclerosis [20], sarcopenia [21], and neurodegenerative diseases such as Alzheimer’s (AD) and Parkinson’s disease [17].

**Fig. 1:**
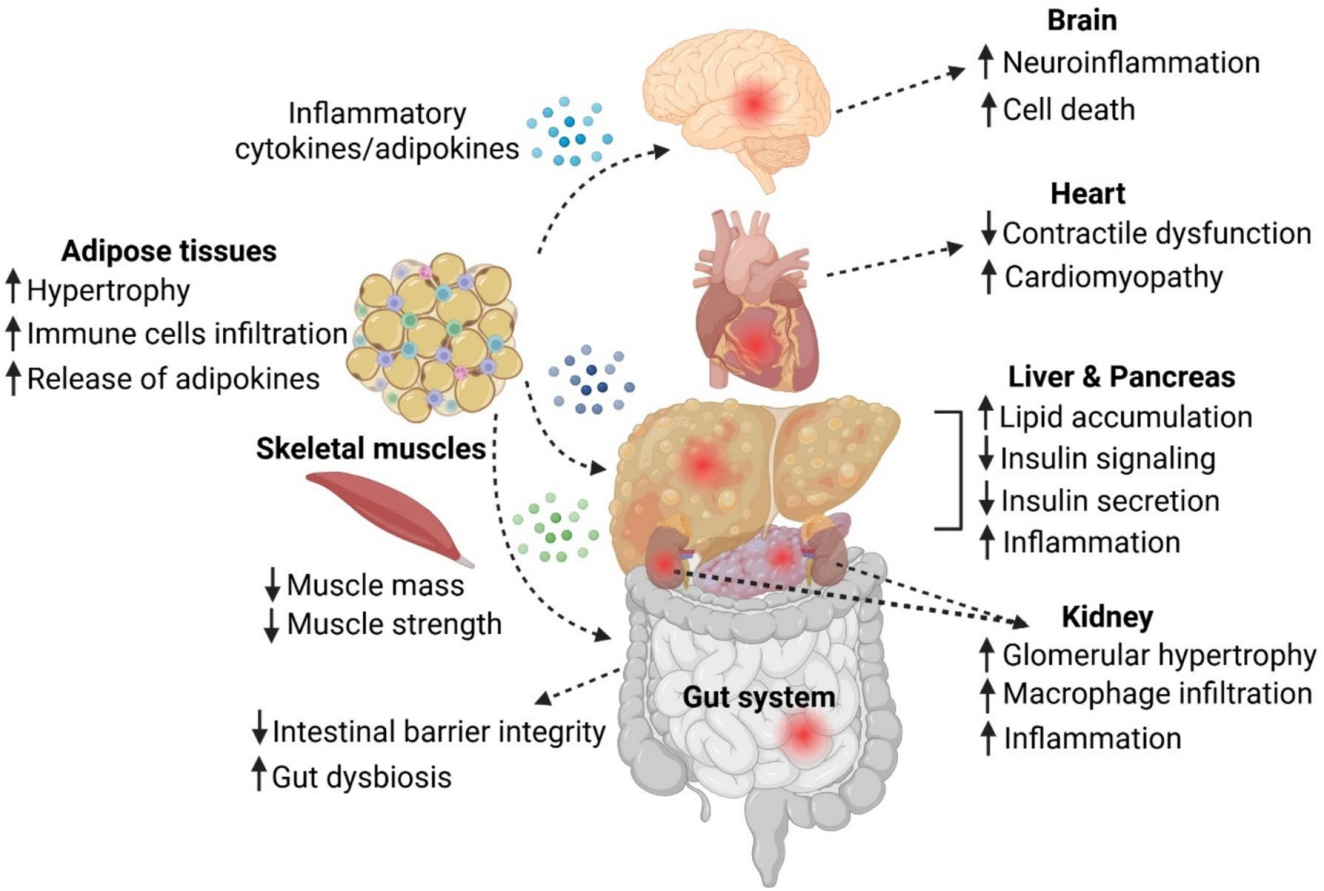
Impact of obesity on various organ systems and tissues. In adipose tissue, obesity induces hypertrophy, immune cell infiltration, and the release of adipokines and inflammatory cytokines, which can propagate systemic inflammation. In skeletal muscle, obesity contributes to reduced muscle mass and strength. In the gut, obesity compromises intestinal barrier integrity and increased gut dysbiosis. In the liver and pancreas, excessive lipid accumulation leads to increased inflammation, impaired insulin signaling, and reduced insulin secretion in the pancreas. In the kidneys, obesity results in glomerular hypertrophy, macrophage infiltration, and heightened inflammation. In the heart, obesity is associated with contractile dysfunction and an increased risk of cardiomyopathy. In the brain, obesity promotes neuroinflammation and neuronal cell death. Furthermore, obesity-driven inflammation and cytokine release facilitate crosstalk between organs, exacerbating systemic metabolic dysfunction. Created with Biorender.com

In this review, we aim to provide insights into the role of autolysosomal dysfunction in obesity-related metabolic dysregulation and inflammation. We will discuss major tissues or organs in the body that are susceptible to obesity, including adipose tissues, skeletal muscles, gut, liver, pancreas, kidney, heart, and brain (Fig. 1). We will also discuss the therapeutic strategies that have been used in preclinical studies to modulate autolysosomal dysfunction in these different tissues or organs under obesity conditions.

### Adipose Tissue

Obesity induces numerous cellular stresses and inflammatory signaling pathways by ectopic accumulation of fat in various tissues, leading to insulin resistance. Among the metabolic organs, adipose tissue (AT) and the liver are especially implicated in energy imbalance and obesity-related pathology [22, 23, 24]. Adipocytes store excessive energy in the form of triglyceride, and AT functions as an endocrine organ that secretes adiponectin, leptin, and pro-inflammatory cytokines such as interleukin-6 (IL-6) and tumor necrosis factor (TNF), and monocyte chemoattractant protein-1 (MCP1). In adipocytes from obese individuals, lysosomal function and autophagy function is suppressed [22, 25, 26, 27]. Autophagy inhibition further leads to increased endoplasmic reticulum stress, which leads to an upregulation of inflammation-related genes, including MCP-1, IL-6, and interleukin-1β (IL-1β) [25]. Pharmacological inhibition of autophagy using 3-methyladenine further exacerbated inflammatory gene expression, underscoring autophagy’s critical role in regulating adipocyte inflammation [22, 25, 26]. Obese adipose-derived stem cells (O-ADSCs) isolated from liposuction specimens of obese donors exhibited weaker Lysotracker Red staining, indicating elevated lysosomal pH and reduced autophagic flux [27]. Moreover, O-ADSCs demonstrated significant mitochondrial dysfunction, including increased fragmentation and reduced organelle size, reduced mitochondrial membrane potential, increased oxidative stress, and impaired energy metabolism [27]. Additionally, these cells showed an increased expression of CD36 (fatty acid translocase) and CD106, markers associated with inflammation [27]. Lysosomal dysfunction has also been observed in AT from obese individuals, as indicated by elevated lysosomal gene expression in mesenteric AT compared to lean subjects [28].

Under conditions of overnutrition, AT undergoes dynamic remodeling, which not only contributes to local inflammation but also promotes systemic inflammation through the recruitment of specialized immune cells that facilitate the clearance of dysfunctional adipocytes and their replacement with new fat cells [24, 29]. Among these immune cells, lipid-associated macrophages (LAMs) play a crucial role in obesity and lipid stress-related inflammation. LAMs selectively express transmembrane 4L six family member 19 (TM4SF19), a lysosomal protein that interacts with the lysosomal V-ATPase, leading to reduced lysosomal acidification. Inactivation of TM4SF19 has been shown to enhance lysosomal acidification, reduce LAM accumulation, accelerate the clearance of apoptotic adipocytes in vitro and in HFD mice, and mitigate inflammation and immune cell infiltration, thereby improving systemic insulin sensitivity [29]. Increased CD36 expression has been observed in human visceral AT, preadipocytes from HFD mice, and 3 T3L1 preadipocytes treated with free fatty acids (FFA) [30, 31]. CD36 overexpression can activate inositol (1,4,5)-trisphosphate receptor 1, leading to lysosomal calcium overload, lysosomal membrane permeabilization (LMP), and decreased V-ATPase activity, resulting in increased lysosomal pH [30, 31]. Additionally, CD36 functions as an inflammatory receptor in adipose tissue [31, 32], with its upregulation by FFAs in preadipocytes triggering increased expression of MCP-1, IL-6, and IL-1β, further propagating systemic inflammation [31].

Exposure to an HFD or FFAs induces LMP in AT, leading to the release of lysosomal proteases such as cathepsin B (CTSB) [30, 33, 34, 35, 36]. Increased cytosolic CTSB contributes to mitochondrial dysfunction [30, 33, 34, 35], which further exacerbates lysosomal acidification impairment [37, 38]. Notably, coincubation of adipocytes with palmitic acid and a selective CTSB inhibitor almost completely prevented the loss of lysosomal acidification induced by palmitic acid [33]. CTSB activation not only promotes adipocyte cell death but also recruits immune cells to AT, triggering the release of inflammatory cytokines that propagate systemic inflammation [22, 30, 33, 34]. In HFD mice, increased LMP has also been associated with the release of cathepsin D, leading to mitochondrial dysfunction and adipocyte cell death [35].

Despite evidence linking lysosomal dysfunction to obesity, few studies have specifically explored strategies to restore lysosomal acidification in adipocytes. Most research has focused on enhancing autophagic function as a whole. For instance, an 8-week treadmill exercise regimen in HFD mice increased autophagic turnover in AT, accompanied by upregulation of LAMP2, a key regulator of autophagosome-lysosome fusion [39]. Similarly, exercise-induced improvements in autophagic machinery were associated with reduced cytosolic CTSB release in HFD mice [40]. However, the impact of exercise on autophagy varies among different adipose depots. While endurance exercise increased autophagy in inguinal and subcutaneous visceral AT, the effect was less pronounced in epididymal AT [41]. Dietary interventions, particularly those rich in unsaturated fatty acids, may also serve as potential therapeutic strategies to restore autophagic function. Long-term consumption of a monounsaturated fatty acid-rich diet upregulated autophagy-related genes BECN1 and Atg7 in subcutaneous AT of obese individuals [42]. Similarly, omega-3 supplementation improved autophagic flux in AT of obese rats [43].

### Skeletal Muscle

Lipotoxicity is a pathological state wherein excessive lipid accumulation in non-adipose tissues perturbs cellular homeostasis and increases cell death [22]. Inadequate lipid droplet biogenesis or storage capacity leads to sustained elevation of circulating free fatty acids, which are ectopically deposited in insulin-sensitive tissues such as skeletal muscle, liver, and pancreas [22]. This aberrant lipid deposition disrupts mitochondrial function, promotes endoplasmic reticulum stress, and activates inflammatory and apoptotic signaling cascades, culminating in insulin resistance and the progression of metabolic disorders [22]. Sarcopenic obesity is characterized by a simultaneous decline in muscle mass and function, accompanied by an increase in adipose tissue mass [44]. This condition is a growing concern among older adults due to its significant health risks, including the development of comorbidities and geriatric syndromes [44]. Maintaining proper protein metabolism is essential for preserving skeletal muscle mass and function. Under obesity, primary human skeletal muscle myotubes exhibit reduced autophagic flux, leading to impaired carbohydrate and lipid metabolism and contributing to muscle atrophy, and insulin resistance [44]. In overweight elderly individuals, elevated expression of autophagy-related proteins such as Atg5, Atg12, LC3-I, LC3-II, and Beclin-1 suggests increased autophagy initiation and autophagosome formation. However, the accumulation of p62 within skeletal muscles indicates impaired autophagic flux, preventing proper degradation and recycling of cellular components [45]. Recent study comparing 49 BMI-discordant monozygotic twin pairs revealed that the more obese co-twins exhibited dysregulated lysosomal metabolic networks, indicative of lysosomal dysfunction, alongside elevated inflammatory pathways [46].

In HFD mice, suppression of autophagy has been observed in gastrocnemius [47] and soleus muscles [48], leading to reduced myofiber cross-sectional area and structural and functional impairments [47, 48]. Treatment of C2 C12 differentiated myotubes with increasing doses of PA resulted in elevated expression of p62 and LC3, signifying autophagic dysfunction. Additionally, GFP-mRFP-LC3-transfected C2C12 myotubes showed an increase in yellow puncta without a corresponding rise in red puncta, indicating impaired autophagosome-lysosome fusion and defective autophagic degradation [47]. Despite growing evidence linking lysosomal dysfunction to skeletal muscle impairment, studies specifically examining how modulating lysosomal acidification affects muscle homeostasis remain limited, underscoring the need for further research in this area.

Exercise interventions have been widely employed to enhance autolysosomal function in skeletal muscle under obesity. Moderate-intensity swim training has been shown to restore autophagic function in the gastrocnemius muscles of HFD mice [49]. Hypoxia training, administered for durations ranging from one to four weeks, increased Beclin-1 and LC3 expression in the gastrocnemius muscle of mice, suggesting enhanced autophagic activity [50]. Additionally, a single bout of exercise stimulation was sufficient to reverse autophagic inhibition in the soleus muscle of mice fed a high-fat, high-sucrose diet [48]. Liraglutide, a GLP-1 receptor agonist, has been shown to enhance autophagy in rat skeletal muscle cells by upregulating sestrin2 via the GLUT4–pAKT–mTOR signaling pathway [51]. Similarly, geniposide activates the GLP-1 receptor and promotes autophagy through the AMPK/mTOR pathway [52]. Moreover, GLP-1 overexpression in skeletal muscle improves glucose metabolism and mitochondrial biogenesis by increasing autophagy [53].

### Gut

Obesity is associated with impaired intestinal barrier function and dysbiosis of the gut microbiota [54]. Autophagy plays an essential role in maintaining gut homeostasis, and dysfunctional autophagy has been linked to gut microbiota imbalance and intestinal dysbiosis [55, 56]. In patients with obesity and T2D, the gut microbiome is enriched in harmful taxa such as *Clostridium leptum, Clostridium coccoides, Enterobacteriaceae*, and *Turicibacter* sp., while beneficial bacteria like *Butyricicoccus* sp., *Akkermansia muciniphila*, and *Faecalibacterium prausnitzii* are significantly reduced [55, 57, 58]. Similarly, in HFD mice, there is an increased abundance of *Butyricimonas*, a reduction in *Akkermansia*, and decreased expression of the tight junction protein occludin, indicating compromised intestinal barrier function [55, 59, 60]. Supporting the role of autophagy, mice with colonic epithelial cell-specific deletion of the autophagy gene *Atg7* display altered fecal microbiota composition, with increased total bacterial load and enrichment of *Clostridium leptum*, *Eubacterium cylindroides*, and *Bacteroides fragilis*, compared to wild-type controls [61]. Overall, gut dysbiosis, particularly the loss of *Akkermansia muciniphila*, is correlated with increased intestinal permeability and endotoxemia-driven systemic inflammation in obesity [62, 63].

Targeting autophagy has emerged as a promising strategy to restore gut barrier integrity and metabolic health [56, 64]. Spermidine, an autophagy inducer, improves insulin resistance in both humans and mice [54]. Mechanistically, spermidine treatment protects gut barrier function by activating autophagy, attenuating apoptosis in colonic and intestinal cells, and increasing the number of mucus-secreting goblet cells and mucin secretion in HFD mice [54]. Similarly, treatment with nuciferine promotes autophagosome formation and autolysosomal fusion in HFD mice, reducing hyperglycemia and intestinal permeability [59]. Furthermore, resveratrol-mediated inhibition of mTORC1, which results in activated autophagy, reduced the abundance of obesity-associated gut microbiota, including *Lactococcus*, *Clostridium XI*, *Oscillibacter*, and *Hydrogenoanaerobacterium*, and alleviated intestinal inflammation in diet-induced obese mice [65]. Probiotics have also shown beneficial effects, helping to rebalance the microbial population by enhancing autophagic function [66, 67].

The gut plays a central role in inter-organ communication, influencing the liver, brain, and other systems [17, 68, 69]. Autolysosomal dysfunction is increasingly seen across these organs, contributing to systemic pathology [17]. In HFD-fed mice, there is an increased *Firmicutes/Bacteroidetes* ratio and reduced *Akkermansia* levels, which were associated with hepatic steatosis. Treatment with apple polyphenol extract reversed these microbiota changes through upregulating autophagy-related genes, leading to reduced hepatic steatosis [70]. In obesity, dysbiosis-driven increases in lipid absorption and circulating metabolites promote mitochondrial and endolysosomal dysfunction in neurons, contributing to AD pathogenesis [71]. The breakdown of the gut barrier facilitates endotoxemia, triggering a pro-inflammatory cascade that disrupts metabolic pathways in the brain [72]. Notably, HFD-induced microbiome alterations can be ameliorated by dimethyl itaconate supplementation, which enriches propionate- and butyrate-producing bacteria. Furthermore, fecal microbiota transplantation from dimethyl itaconate-treated mice improves cognitive function and hippocampal synaptic structure, indicating restoration of brain metabolic health [73].

### Liver

During obesity, excessive FFAs surpass the liver’s capacity to store them as lipid droplets, leading to lipotoxicity, increased liver inflammation, and impaired hepatic function [74]. Liver biopsy specimens from patients with MASLD show significantly reduced expression of lysosomal cathepsins (e.g., CTSB, CTSD, and CTSL) and increased p62 accumulation, indicative of autolysosomal dysfunction [75]. Elevated serum alanine aminotransferase (ALT) levels further suggest hepatic inflammation [75]. Additionally, liver biopsies from morbidly obese patients undergoing bariatric surgery exhibit impaired autophagy, along with increased hepatic apoptosis and pyroptosis [76]. In three different murine models of MASLD, reduced numbers of acidic organelles and lower levels of the lysosomal enzyme CTSD point to defective lysosomal acidification [77].

In hepatocytes treated with PA, elevated lysosomal pH disrupts autophagic degradation and mitochondrial function, leading to lipid droplet accumulation and increased insulin resistance [14]. PA-treated primary hepatocytes also exhibit downregulation of lysosomal V-ATPase subunit ATP6V1A and LAMP1, indicating impaired lysosomal acidification [78]. Furthermore, TFEB expression is reduced in the livers of HFD mice, signifying compromised lysosomal function [78, 79]. Obesity-induced lysosomal dysfunction is exacerbated by increased nitric oxide production, which promotes nitrosative stress and S-nitrosylation, further impairing lysosomal function and exacerbating hepatic inflammation in HFD mice [80]. Treatment of cultured hepatocytes with both oleate and PA results in increased lipid droplet accumulation and impaired autophagic clearance [81].

Exercise has been shown to enhance lysosomal function, as indicated by increased LAMP1 and LAMP2 levels in HFD mice [82]. Voluntary wheel running [83] and swim training [84] exercises both improved autophagic activity and reduced hepatic lipid accumulation in MASLD mouse models [85]. Dietary supplementation with docosahexaenoic acid (DHA) and eicosapentaenoic acid (EPA) mitigated lipid accumulation and cell death in FFA-exposed L02 liver cells [86]. The combined effects of DHA supplementation and aerobic exercise synergistically enhanced autophagic function in HFD mice [87]. In HFD mice, oleic acid supplementation upregulated lysosomal TFEB expression and enhanced lysosomal acidification [88]. Treatment with phillygenin, a lignan derived from *Forsythia suspensa*, promoted TFEB dephosphorylation, enhancing lysosomal biogenesis and attenuating NLRP3 inflammasome activation, thereby reducing liver inflammation [78]. Similarly, baicalein supplementation in HFD mice increased the expression of lysosomal V-ATPase V1 subunits, improving lysosomal acidification and function while reducing LMP and hepatic inflammation [89]. Tetrahydrocurcumin similarly upregulated lysosomal biogenesis through TFEB nuclear translocation, mediated via mTORC1 inhibition [90]. The GLP-1 receptor agonist liraglutide restores autophagic activity in HFD mice by promoting lysosomal biogenesis and function [91], and enhances the expression of cytosolic lipolysis-related proteins, thereby reducing intracellular lipid accumulation [92]. Semaglutide, an incretin mimetic, increased autophagic function and reduced lipid accumulation in steatotic hepatocytes [93]. Finally, recent work has shown that lysosome-targeting acidic nanoparticles (NPs) composed of poly(tetrafluorosuccinate-co-succinate) ester (PEFSU NPs) successfully increased lysosomal acidification and autophagic degradation, reducing lipid droplet accumulation and hepatic inflammation in HFD mice [14].

### Pancreas

When the pancreas is exposed to high amounts of lipid in obesity, impairment of autophagic flux and lysosomal function are found in the pancreatic β-cells, as well as reduced insulin secretion and cellular viability [18, 37, 38]. In islets isolated from T2D human subjects, there is downregulation of lysosomal V-ATPase subunit genes *ATP6V1B2*, *ATP6AP2*, *ATP6V1A*, *ATP6V1H*, *ATP6V1G1*, *ATP6V0E1* and *ATP6V0B* [18]. In HFD mouse model of T2D, autolysosomal dysfunction was present in the mice pancreas [18]. Studies in other diabetic mouse models have shown a reduction in lysosomal subunit ATP6V1A in mouse islets [94, 95]. Similarly, in INS-1 832/13 rat insulinoma cells treated with palmitate, autolysosomal function is impaired, consequently leading to reduced glucose-stimulated insulin secretion [18, 96, 97]. Expression levels of inflammatory markers TNF, IL-1β and IL-6 were also increased in INS-1 cells under palmitate [98]. While underexplored, a study has shown that pancreatic alpha cells from HFD mice show changes in protein degradative pathways, including lysosomal pathways [99].

Lysosome-targeted nanoparticles have emerged as a promising strategy for restoring lysosomal acidification defects in T2D cellular and animal models. These include photo-activated nanoparticles that release acids upon UV-light exposure to lower lysosomal pH [96], and poly(tetrafluorosuccinate)-based nanoparticles (TFSA NPs), which release acidic components within the lysosomal lumen to restore acidification in pancreatic β-cells [18]. Quercetin, a natural flavonoid, has been shown to reverse lysosomal dysfunction and enhance autophagosome-lysosome fusion in pancreatic β-cells under palmitate treatment [100]. Additionally, supplementation with unsaturated fatty acids such as docosahexaenoic acid has been reported to enhance autophagy in palmitic acid-treated mouse insulinoma 6 cells via modulation of the mTOR/GPR120 axis [101]. Omega-3 fatty acid enrichment has also been demonstrated to improve autophagic flux in pancreatic β-cells of STZ-treated fat-1 mice, thereby reducing diabetes-related β-cell damage [102].

Lifestyle interventions such as physical exercise have been explored as potential strategies to restore pancreatic function. High-intensity training has been found to enhance pancreatic β-cell function in both T2D patients [103] and healthy individuals following a high-fat meal [104]. Additionally, physical exercise has been shown to improve islet morphology, increase β-cell number, and reduce β-cell apoptosis in obese rats [105], though the precise mechanisms regulating β-cell activity remain unclear. Another lifestyle intervention, caloric restriction, has been demonstrated to enhance autophagic flux in obese mice and rats [106, 107]. Intermittent fasting (IF) has also been shown to reduce the accumulation of autophagic substrates and increase TFEB expression, indicating improved lysosomal function [108]. Furthermore, IF promotes β-cell proliferation, potentially through activation of the autophagy–lysosome pathway and upregulation of Neurogenin-3 (Ngn3) expression [109, 110]. Notably, the protective effects of IF, including inhibition of β-cell mortality and promotion of Ngn3 expression, were absent in obese mice with lysosomal deficiencies or autophagosome dysfunctions, underscoring the critical role of the autophagy–lysosome pathway in these processes [108].

### Kidney

The systemic inflammation driven by increased adipokine secretion from adipose tissue exacerbates lipid accumulation in multiple organs, including the kidneys, contributing to glomerular hypertrophy and segmental sclerosis, which impairs renal function [111, 112]. Studies in both human and experimental models have demonstrated that impaired lysosomal function and disrupted autophagic flux play a crucial role in obesity-related kidney disease by promoting lipotoxicity and inflammation [112, 113]. In patients with chronic kidney disease (CKD), a higher body mass index has been linked to increased vacuolation and decreased nuclear TFEB levels in proximal tubules, indicating autophagy dysregulation [112]. Additionally, kidney biopsy samples from obese CKD patients revealed LAMP1-positive vacuoles and p62 accumulation in renal proximal tubular epithelial cells (PTECs), further suggesting impaired autolysosomal function [19, 112]. In vitro studies using palmitate-treated PTECs have also shown reduced lysosomal acidification, reinforcing the role of autolysosomal dysfunction in obesity-associated renal pathology [19]. This reduction in autolysosomal activity has been linked to mitochondrial dysfunction, increased macrophage infiltration, and inflammasome activation, all of which contribute to renal fibrosis and disease progression [19].

In patients with diabetic nephropathy, renal biopsy tissues exhibited reduced TFEB expression, accompanied by lipid accumulation and increased apoptosis, indicating lysosomal and autophagic dysfunction [114]. Similarly, HFD rats displayed lower nuclear TFEB levels compared to those on a low-fat diet, further suggesting impaired lysosomal function [114]. Additionally, HFD rats showed autophagy suppression alongside elevated serum cystatin C levels and increased urinary N-acetyl-β-d-glucosaminidase activity, both markers of kidney injury [115]. Proximal tubular epithelial cell–specific mice fed on HFD have increased phospholipid accumulation in enlarged lysosomes indicative of lysosomal dysfunction, and reduced autophagic function [112]. In HK-2 cells, palmitate treatment induced TFEB phosphorylation at Ser211, which impaired its nuclear translocation, thereby disrupting TFEB-mediated lysosomal biogenesis and function [115]. Another study using the same cellular model demonstrated that lysosomal V-ATPase subunit ATP6V1D protein expression was significantly reduced, indicating a decline in lysosomal acidification and function [114]. Moreover, palmitate exposure was found to promote TFEB dephosphorylation and nuclear translocation by inhibiting the mTOR pathway in a Rag GTPase–dependent manner, further linking autophagic dysfunction to metabolic stress in renal cells [112].

Few studies have specifically targeted lysosomal acidification and function within the kidney, with most focusing on enhancing autophagic activity more broadly. Sodium-glucose co-transporter inhibitors have been shown to mitigate autophagy suppression in the kidney by reducing mTOR expression in HFHS mice, thereby restoring autophagic function [116]. Epigallocatechin-3-gallate (EGCG) has demonstrated the ability to enhance autophagy in palmitate-treated HK-2 cells, while also increasing AMP-activated protein kinase (AMPK) phosphorylation in the kidneys of HFD rats and palmitate-treated HK-2 cells, contributing to improved autophagic activity [115]. Additionally, supplementation with eicosapentaenoic acid, a polyunsaturated fatty acid, has been found to rescue autophagy impairment in renal PTECs [117]. Exercise-based interventions have also shown promise, with endurance exercise in obese mice activating ULK-1, increasing Beclin-1 and LAMP1 levels, and promoting AMPK activation, leading to enhanced autophagic and lysosomal function in kidney tissues [118]. Similarly, treadmill exercise in obese rats activated AMPK while suppressing mTOR signaling in kidney tissues, further supporting the role of exercise in improving autophagic function [119].

### Heart

Obese and overweight individuals with AT hypertrophy exhibit significant alterations in cardiac structure and function, increasing the risk of cardiomyopathy, hypertrophy, atrial fibrillation, and arrhythmia. AT hypertrophy is associated with the secretion of atherogenic cytokines that impair autolysosomal function in cardiac cells [20]. In the right atrial appendage cardiac tissues of obese individuals, TFEB expression is significantly reduced compared to healthy individuals, indicating compromised lysosomal acidification and function [120]. Similarly, in mice fed a HFD or a HFHS diet, cardiomyocytes show decreased TFEB content [120, 121, 122], along with a significant increase in diacylglycerol and triacylglycerol levels, indicating lipid metabolism dysregulation and cardiomyocyte cell death [123]. Additionally, cardiomyocytes in obese models exhibit reduced lysosomal V-ATPase activity and elevated lysosomal pH, indicative of impaired lysosomal acidification [124]. In both HFD mice and palmitate-treated H9C2 cardiomyocytes, lysosomal acidification and autophagosome clearance are significantly suppressed [125]. This dysfunction in lysosomal acidification within cardiomyocytes contributes to a marked reduction in sarcomere shortening, leading to contractile dysfunction and the progression of cardiomyopathy [124, 126].

Palmitate treatment of cardiomyocytes increased s-nitrosylation of a highly conserved cysteine residue (Cys-277) of the ATP6V1A1 subunit, which could impair V-ATPase function and reduce lysosomal acidification [125]. Similar phenomena were also observed in cardiac cells including induced pluripotent stem cell cardiomyocytes, HL-1 and aRCM, where palmitate treatment reduced lysosomal V-ATPase function through dissociation of the V-ATPase [124, 126, 127]. In addition, palmitate induced activation of NADPH oxidase (Nox-2), which leads to superoxide production that impairs lysosome acidification and enzyme activity [125]. Exposure to palmitate acid has also been shown to decrease TFEB expression in various cell types including H9C2 [120, 121, 122, 123], HL-1 [123], adult mouse cardiomyocytes [123] and neonatal rat cardiomyocytes [121], as well as reduce lysosomal enzyme activity [121, 123].

Therapeutic strategies targeting lysosomal acidification have shown promise in restoring autophagic function in cardiac cells. Poly(lactic-co-glycolic) acid (PLGA) NPs have been utilized to reverse lysosomal de-acidification in palmitate treated H9C2 cardiomyocytes, aiding in cellular homeostasis [128]. Additionally, eicosapentaenoic acid (EPA) supplementation has been found to activate AMPK, leading to mTOR inhibition and ULK1 activation, thereby promoting autophagy initiation in palmitate-treated H9C2 cardiomyocytes [129, 130] Canagliflozin, a sodium-glucose cotransporter 2 inhibitor, has demonstrated efficacy in inhibiting mTOR and enhancing autophagic activity in both HFD/STZ-induced mouse hearts and palmitate-treated HL-1 cardiomyocytes [131]. Regular aerobic exercise has also been shown to activate AMPK in cardiac muscle, supporting autophagic processes [132, 133]. A recent study highlighted that rhythmic handgrip exercises improved key autophagy markers, including increased Beclin1, LC3B, Atg3, and LAMP2 levels, along with reduced p62 levels in endothelial cells from the human radial artery, suggesting enhanced autophagic function [134]. Furthermore, calorie restriction has been associated with increased autophagosome formation in heart muscle, indicating improved autophagic flux [135]. Intermittent fasting has also been reported to stimulate lysosomal function in cardiac tissues, further supporting its potential as a metabolic intervention [136].

### Brain

Obesity-induced metabolic disorders can result in an increase in systemic inflammation, which can potentially propagate and induce neuroinflammation [17, 137]. There are increased levels of palmitic acid in the plasma of obese individuals. Hypothalamus, a brain region with a key role in the regulation of energy balance, food intake, insulin sensitivity and glucose homeostasis, has the greatest susceptibility to changes in the amount of palmitic acid derived from diets [138]. In rodent models of obesity, the hypothalamus has the highest amount of palmitic acid accumulation under HFD [138, 139, 140]. Mice with exposure to 16 weeks of HFD have decreased protein levels of Beclin-1 and LC3-II along with increased levels of p62, indicating reduced autophagic flux in the hypothalamus [141]. In addition, there is increased inflammation and ER stress as indicated by increased IKKβ/NF-kB activation and EIF2α expression [141]. This trend is also observed in mice hippocampus [142, 143, 144] where HFD feeding induced accumulation of LC3, Atg3, Beclin1 and p62 in the hippocampus indicative of reduced autophagic flux, leading to hippocampal amyloidosis [142]. HFD feeding also increased the activation of microglia and astrocytes, and increased NLRP3 inflammasome levels, leading to neuroinflammation [144].

Exposure of embryonic mouse hypothalamus cell line, N43-5, to palmitate resulted in the activation of free fatty acid receptor 1 leading to decreased autophagic flux, leading to insulin resistance [139]. In another study, palmitate treatment to N43-5 resulted in LC3 accumulation and lowered its co-localization with LAMP1 indicating impairment of autophagosome-lysosome fusion, potentially due to reduced lysosomal acidification, leading to increased neuroinflammation [138]. Palmitate treatment to other neuronal cell lines such as CLU-189 [141] and BV2 [142] have also shown reduction in autophagic flux. In cholesterol-enriched SH-SY5Y cells and primary neurons, high intracellular cholesterol levels stimulated mitochondrial PINK1 accumulation and mitophagosomes formation, indicative of reduced autophagic flux [145]. This trend is also seen in an AD mouse model [145].

Dietary supplementation with unsaturated fatty acids and exercise have demonstrated restorative effects in brain cells under obesity or lipotoxicity. Using Fat-1 transgenic mice which produces endogenous levels of n-3 polyunsaturated fatty acids, it was shown that there is an increased expression of ATG7 and LC3II, as well as reduced p62, indicative of increased autophagic flux in the hypothalamus [146]. Oleic acid ameliorates the impairment in fusion between autophagosomes and lysosomes within mHypoE-43/5 neuronal cells under palmitate treatment [147]. Autophagic function was also restored in the brain of obese rats after weight maintenance following short-term caloric restriction, long-term caloric restriction, and long-term exercise [148]. In addition, GLP-1 mimetic exendin-4 enhances autophagy and protects against T2D-induced apoptosis. It increases cortical GLP-1 and IGF-1 levels in mice and activates PKA and PI3K/Akt signaling pathways, leading to upregulation of autophagy and lysosomal markers such as Atg7 and LAMP-1, thereby reducing apoptotic cell death [149]. Other therapeutic agents that restore autophagic and cellular function in CNS cells under obesity have been reviewed elsewhere [150, 151].

## Summary and Future Directions

**Fig. 2:**
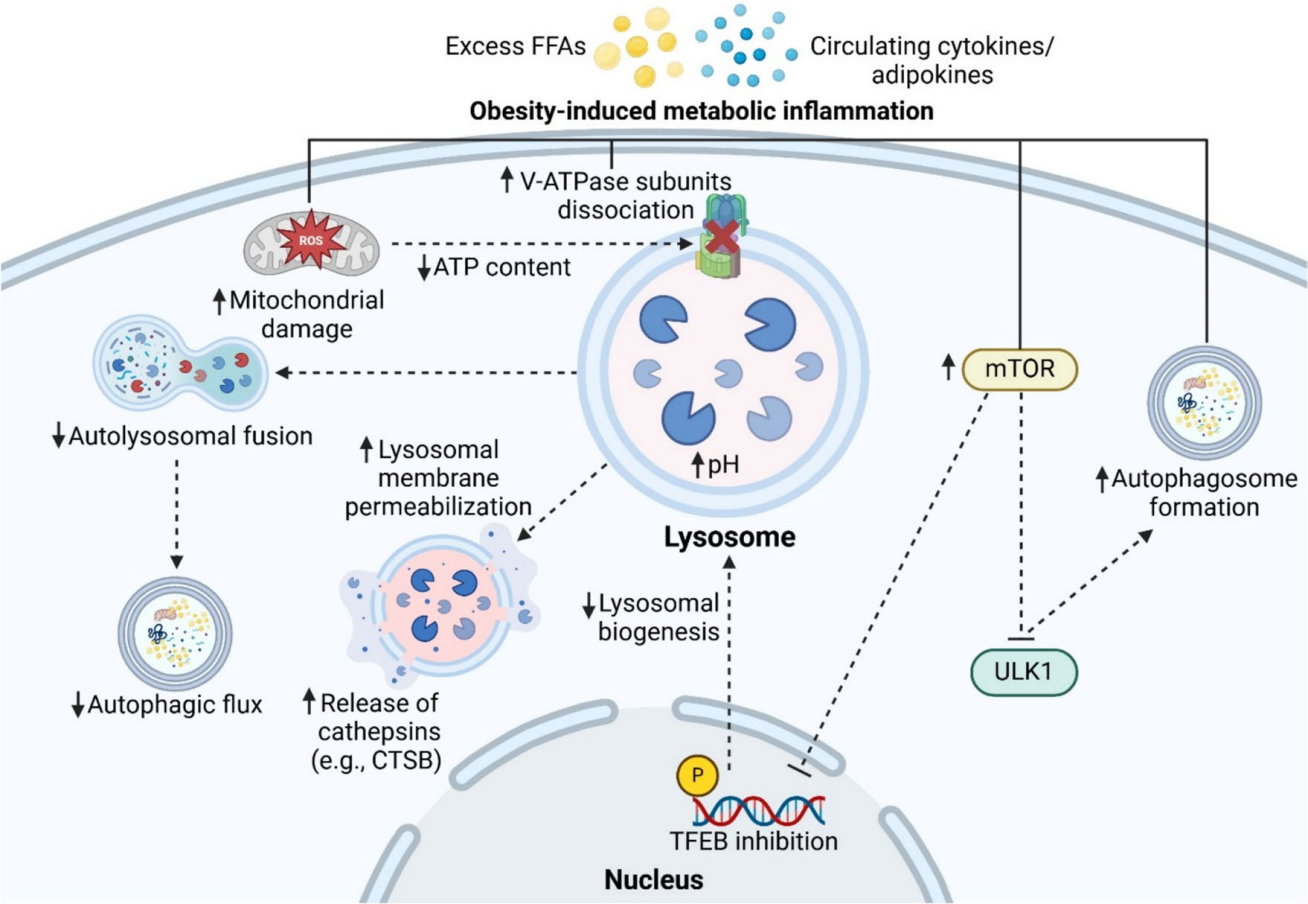
Key mechanisms by which obesity contributes to lysosomal acidification dysfunction. Under obesity-induced metabolic inflammation, excess free fatty acids (FFAs), cytokines, and adipokines disrupt lysosomal function by promoting the dissociation of lysosomal V-ATPase subunits, leading to increased lysosomal pH. Mitochondrial damage further exacerbates this dysfunction by reducing ATP production, limiting the energy supply required for lysosomal V-ATPase activity. Elevated lysosomal pH can induce lysosomal membrane permeabilization, resulting in the release of lysosomal cathepsins (e.g., CTSB) into the cytosol, triggering inflammation and cell death. Additionally, impaired autophagosome-lysosome fusion reduces autophagic flux. Excess lipids and cytokines also activate mTOR, which inhibits the ULK1, thereby suppressing autophagosome formation. Furthermore, mTOR activation suppresses TFEB activity, reducing lysosomal biogenesis and contributing to increased lysosomal pH. Created with Biorender.com

Obesity is a significant and growing global health challenge with widespread implications for metabolic disorders, including T2D, MASLD, cardiovascular diseases, kidney disease, neuroinflammation, and neurodegeneration. Emerging research highlights the intricate link between obesity and autolysosomal dysfunction across multiple organs, such as adipose tissue, liver, pancreas, kidney, heart, and skeletal muscle. In adipose tissue, obesity-induced autophagic suppression and lysosomal dysfunction drive inflammation and metabolic disturbances. Similarly, in the liver, these impairments exacerbate lipotoxicity and insulin resistance, while in the pancreas, dysregulated autophagy affects insulin secretion and pancreatic β-cell viability. In the kidneys, obesity disrupts autophagic flux, contributing to renal dysfunction, whereas in the heart, defects in lysosomal acidification impair cardiac function. Skeletal muscles also experience reduced autophagic activity, compromising muscle function and structure. Obesity has also been shown to compromise the integrity of the intestinal epithelial barrier [56], and enhancing autophagic function may help restore barrier function [56, 152]. The shared mechanisms by which obesity disrupts lysosomal acidification and autophagy function in different organs are illustrated in Fig. 2.

**Fig. 3:**
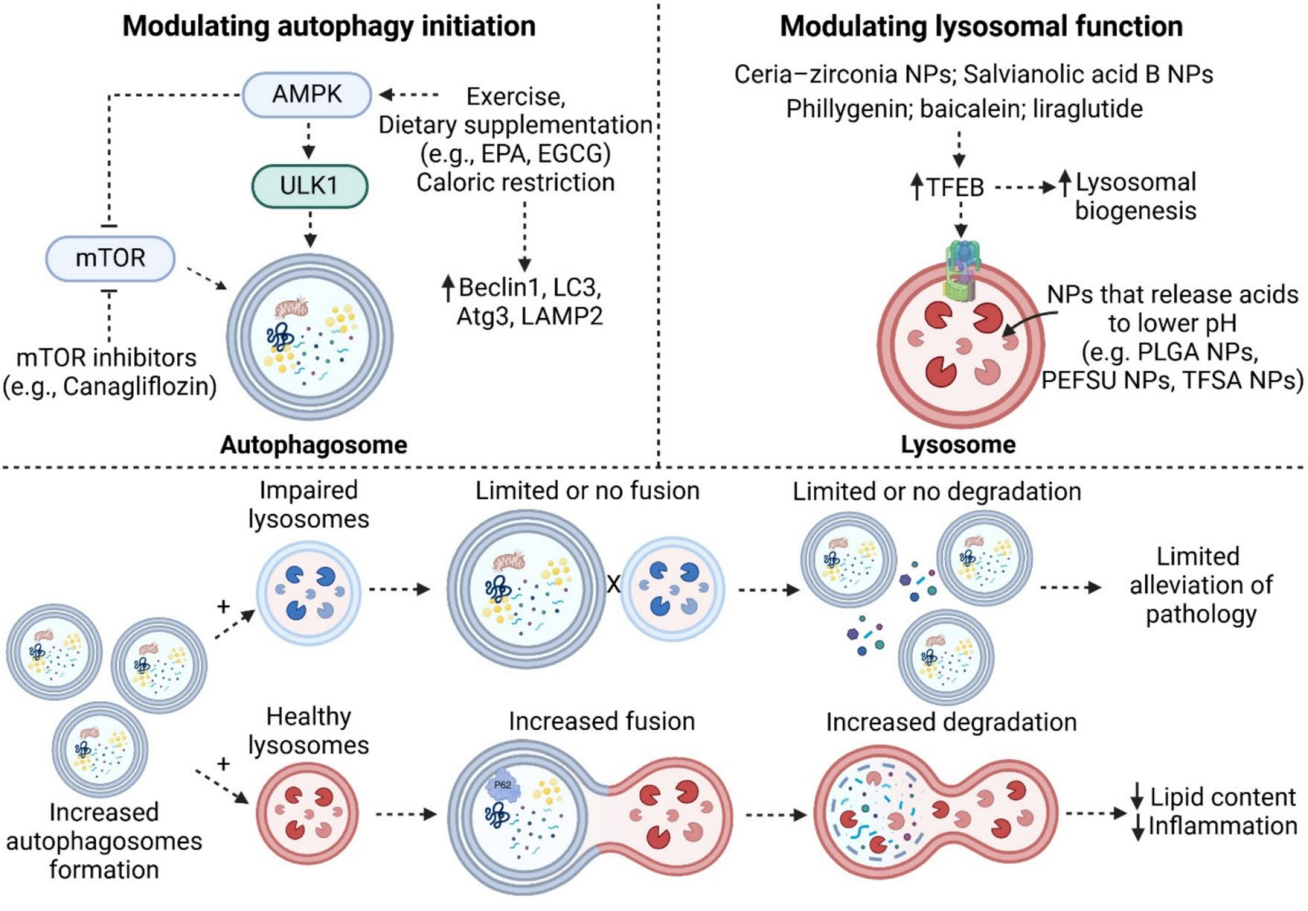
Summary of therapeutic agents modulating autophagy initiation and lysosomal function. Therapeutic strategies targeting autophagy initiation primarily involve exercise, dietary supplementation (e.g., EPA, EGCG), and caloric restriction, which enhance AMPK activation. This, in turn, promotes ULK1 activation, leading to increased autophagosome formation while simultaneously inhibiting mTOR to further enhance autophagy. Pharmacological agents such as canagliflozin inhibit mTOR to induce autophagy. To restore lysosomal function, nanoparticles such as ceria-zirconia and salvianolic acid B NPs, as well as compounds like phillygenin, baicalein, and liraglutide, have been shown to upregulate TFEB, thereby enhancing lysosomal biogenesis. Additionally, nanoparticles that are designed to release acidic components (e.g., PLGA NPs, PEFSU NPs, TFSA NPs) to lower lysosomal pH can improve lysosomal acidification and function. It is important to note that while increasing autophagy initiation leads to greater autophagosome formation, this alone is insufficient if lysosomal function remains impaired. Without functional lysosomes, autophagosomes cannot fuse and degrade their contents effectively. Therefore, therapeutic approaches should target both autophagy initiation and lysosomal function to achieve optimal cellular restoration. Created with Biorender.com

Therapeutic strategies aimed at enhancing autophagy and lysosomal function hold promise for mitigating obesity-related organ dysfunction. Interventions such as exercise, dietary modifications, and emerging treatments, including nanoparticles and pharmacological agents, have shown potential in restoring autophagic activity and lysosomal acidification. For example, exercise has been demonstrated to improve autophagic flux and lysosomal function across multiple tissues [153, 154], while dietary supplementation with unsaturated fatty acids help counteract autophagic impairments [155, 156]. Additionally, novel agents such as nanoparticles designed to enhance lysosomal acidification have shown significant potential in restoring autophagic function and alleviating cellular dysfunction and inflammation [14, 18]. However, fewer studies have investigated the application of nanoparticles for restoring autolysosomal function in adipose tissue, kidneys, skeletal muscles, and brain cells in the context of obesity. Ceria–zirconia NPs [157, 158] and salvianolic acid B NPs [159] have been shown to enhance autophagic flux by promoting TFEB nuclear translocation and improving lysosomal function in kidney models of Fabry disease and acute kidney injury, respectively, suggesting their potential application in obesity-induced kidney disease. Furthermore, nanoparticles have demonstrated the ability to rescue autolysosomal dysfunction in neurodegenerative disease models [160, 161, 162, 163, 164], highlighting their possible therapeutic use in obesity-induced neurodegeneration. Therapeutic agents that restore lysosomal acidification and autophagic function across different organs are summarized in Fig. 3. While increased autophagy initiation can enhance autophagosome formation, impaired lysosomal function prevents autophagosome-lysosome fusion, which can lead to incomplete degradation, and inability to fully mitigate cellular dysfunction. Therefore, future therapeutic strategies could focus on both enhancing autophagy initiation and restoring lysosomal function to ensure effective degradation and cellular homeostasis. In addition, as obesity induces chronic low-grade metabolic inflammation across various tissues, future strategies could incorporate targeting inflammatory pathways to mitigate systemic inflammation and its progression [165, 166, 167, 168].

In summary, elucidating the mechanistic connections between obesity and autophagy provides critical insights into novel therapeutic avenues. Importantly, there are complex multidirectional interactions and inter-organ crosstalk (e.g., gut-liver-brain axis) among affected tissues [169, 170, 171]. This interconnected dysfunction can further amplify systemic inflammation, insulin resistance, and organ-specific impairments, perpetuating a cycle of cellular and metabolic dysfunction [17, 169, 170, 171]. Future studies into this could utilize tools like multi-omics to elucidate the heterogeneity in contribution by different organs and tissues [172, 173, 174]. Integration of these approaches allows for the development of more effective treatments for obesity and its associated complications, leading to improved public health outcomes.

## Key References

- Wang B, Zhang G, Hu Y, Mohsin A, Chen Z, Hao W, et al. Uncovering impaired mitochondrial and lysosomal function in adipose-derived stem cells from obese individuals with altered biological activity. Stem Cell Res Ther. 2024; 15:12.
  - Adipose-derived stem cells isolated from liposuction specimens of obese donors exhibited autolysosomal impairment.
- Lo CH, O’Connor LM, Loi GWZ, Saipuljumri EN, Indajang J, Lopes KM, et al. Acidic Nanoparticles Restore Lysosomal Acidification and Rescue Metabolic Dysfunction in Pancreatic β-Cells under Lipotoxic Conditions. ACS Nano. 2024; 18:15452–67. https://doi.org/10.1021/acsnano.3c09206
  - Mouse pancreatic cells show reduced lysosomal V-ATPase content indicative of lysosomal acidification impairment.
- Choi C, Jeong YL, Park K-M, Kim M, Kim S, Jo H, et al. TM4SF19-mediated control of lysosomal activity in macrophages contributes to obesity-induced inflammation and metabolic dysfunction. Nat Commun. 2024; 15:2779. https://doi.org/10.1038/s41467-024–47108-8
  - Lipid-associated macrophages express transmembrane 4L six family member 19 (TM4SF19) that interacts with lysosomal V-ATPase and reduce lysosomal acidification.
- Zeng J, Acin-Perez R, Assali EA, Martin A, Brownstein AJ, Petcherski A, et al. Restoration of lysosomal acidification rescues autophagy and metabolic dysfunction in non-alcoholic fatty liver disease. Nat Commun. 2023; 14:2573. https://doi.org/10.1038/s41467-023–38165-6
  - Chronic exposure to lipids induces autolysosomal dysfunction in liver cells, leading to reduced mitochondrial function, insulin resistance and liver inflammation.
- Wang S, Schianchi F, Neumann D, Wong L-Y, Sun A, van Nieuwenhoven FA, et al. Specific amino acid supplementation rescues the heart from lipid overload-induced insulin resistance and contractile dysfunction by targeting the endosomal mTOR–v-ATPase axis. Mol Metab. 2021; 53:101293.
  - Lysosomal acidification impairment in cardiomyocytes contributes to cellular dysfunction and progression of cardiomyopathy.
- Cai M, Jiang X, Wei Y, Wen R, Du X. Role of TFEB-autophagy lysosomal pathway in palmitic acid induced renal tubular epithelial cell injury. Biochem Biophys Res Commun. 2024; 696:149472.
  - Reduced TFEB expression is seen in renal biopsy tissues from patients with diabetic nephropathy, indicative of lysosomal and autophagic dysfunction.

## Data Availability

No datasets were generated or analysed during the current study.
